# Exploring Temporomandibular Disorders (TMDs) and Occlusion Debate in Dentistry: Biting Into Controversy

**DOI:** 10.7759/cureus.61108

**Published:** 2024-05-26

**Authors:** Kumari Monika, Amit Reche, Shweta Tagore

**Affiliations:** 1 Public Health Dentistry, Sharad Pawar Dental College and Hospital, Datta Meghe Institute of Higher Education and Research, Wardha, IND

**Keywords:** orthodontics, malocclusion, occlusion, aetiology, tmds

## Abstract

Some conditions known as temporomandibular disorders (TMDs) affect surrounding muscles and jaw joints. In dentistry, there has been discussion and research on the connection between TMDs and occlusion, which is how the upper and lower teeth meet. Although some dental experts have proposed a direct link between TMDs and occlusion, the specifics of this relationship are still unclear and have many facets. More particularly, the research facets of "occlusion" remain one of the most contentious subjects in TMDs. This abstract aims to provide an overview of TMDs and occlusion, summarizing the key points from the literature. The etiological factors contributing to the TMDs, including occlusal, psychological, and hormonal factors, are also analyzed. The second part of the article includes the concept of malocclusion, emphasizing its significance in masticatory function and overall health. Anterior open and posterior open bites and the potential influence of occlusal factors on TMDs are elucidated.

## Introduction and background

Temporomandibular disorders (TMDs) have cumbersome, heterogeneous causes. There are numerous potential causes of a TMD [1]. The importance of occlusion as an etiological component in the emergence of TMDs has been a heated dispute among dentists for ages. Because most arguments for causation are based on anecdotal evidence rather than scientific data, the function of occlusion in the development of TMD remains debatable. Since the "occlusal question" has not been answered, the occlusion-TMD topic is still frequently the subject of conjecture [2].

Surgical, periodontic, prosthodontic, and orthodontic patients frequently exhibit occlusal change [3]. Occlusal changes can be caused by missing teeth, correcting malocclusions, or extracting teeth, which dentists often do. However, after occlusal modification, what will happen? According to clinical evidence, occlusion-altering procedures may cause immediate discomfort in patients. After a few days, this discomfort may go away or get worse, resulting in complaints of ongoing pain in the functional complex of tissues and organs housed within the oral and craniofacial cavities, as well as the development of TMDs, which are a body of persistent pain state affecting the masticatory muscles, the temporomandibular joint (TMJ) and other tissues, and structures [4]. Ages 20 to 40 are the most common ranges for temporomandibular dysfunction in people [5]. Biomechanical, neuromuscular, biopsychosocial, and neurobiological may be the components of TMDs [6]. These components are divided into three categories to highlight their roles in the development of TMD: aggravating (parafunctional, hormonal, or psychosocial factors), initiating (trauma or repetitive adverse loading of the masticatory system), and predisposing (structural, metabolic, and psychological conditions) [7]. Some studies show that occlusion plays a very central role in the etiology of TMD [8]. However, many TMD specialists hold contradictory opinions [9], and a variety of dental procedures, including joint orthodontic therapy, have been implicated as TMD triggers [10]. However, in the most recent investigations, no differences were found between patients with malocclusion and those with normal occlusion [11], as well as between those who had undergone orthodontic treatment and those who had not [12]. Bruxism, exogenous estrogen, orthodontic instability, occlusal abnormalities, joint laxity, and micro and macro trauma are among the conditions' causes. TMD symptoms include TMJ pain that can radiate or refer to nearby or distant structures; otalgia, tinnitus, or both in the absence of the aural disease; and clicking, popping, or crepitus of the TMJ on any movements in which the joint is locked [13]. Durham et al. stated that some patients present primarily due to reported painless clicking (disc displacement with reduction). Others describe a failure to open the mouth widely (disc displacement with reduction with intermittent, limited opening), a prolonged closed lock (disc displacement without reduction with limited opening), or repeated dislocation of the TMJ. Recurrent painful (closed) locking is also described.

## Review

Search methodology

**Figure 1:**
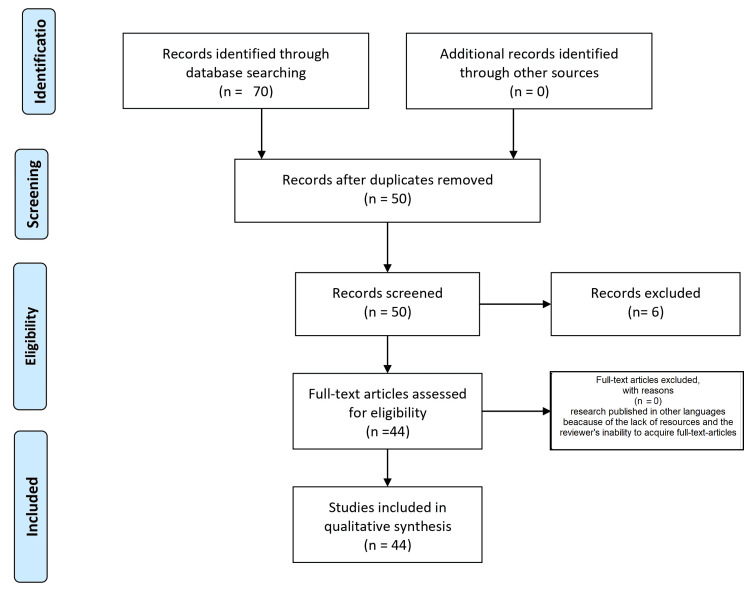
PRISMA flow diagram of the included study. PRISMA: Preferred Reporting Items for Systematic Reviews and Meta-Analyses (pubmed.ncbi.nlm.nih.gov; https://scholar.google.com/)

We conducted a review through PubMed and Google scholar using a combination of keywords, such as "temporomandibular disorder," "TMD," "occlusal factors," "psychological factor," "hormonal factor," "secondary occlusal changes," "anterior open bite," "unilateral posterior open bite," ((( "temporomandibular disorder" [Title/Abstract]) OR ("temporomandibular disorder" [MeSH Terms]), (("TMD" [Title/Abstract]) OR ("TMD" [MeSH Terms]), ("Occlusal factor" [Title/Abstract]) OR ("Occlusal factor" [MeSH Terms], ("Psychological factor" [Title/Abstract]) or ("Hormonal factor" [Title/Abstract]) OR ("Hormonal factor" [MeSH Terms], ("Secondary occlusal changes" [Title/Abstract]) OR ("Secondary occlusal changes" [MeSH Terms], ("Anterior open bite" [Title/Abstract]) OR ("Anterior open bite" [MeSH Terms],("Unilateral posterior open bite" [Title/Abstract]) OR ("Unilateral posterior open bite" [MeSH Terms]. For inclusion, studies in both published reports in English were considered. We excluded research published in other languages because of the lack of resources and the reviewer's inability to acquire full-text articles. Figure 1 represents the search study of the articles included in the review.

Search Results

A bibliographic search of PubMed and Google Scholar databases identified 70 relevant articles, of which 50 were duplicates. A manual search was done on Google Scholar, where the first 10 pages were considered. The first stage of screening resulted in excluding six articles based on title and abstract screening. After title and abstract screening, 44 articles remained and were read in full. Articles were eliminated based on the inclusion and exclusion criteria; the reasons for exclusion are included in the PRISMA flowchart (Figure 1).

TMDs

TMJ dysfunction and associated pain are collectively referred to as TMDs. Regional facial and preauricular pain, restrictions in jaw mobility, and TMJ noises during jaw movement are among its most prevalent symptoms [14]. Costen was the initial one to identify the warning mark of TMDs in 1934 [15]. The earliest description of TMD was made in 1887 by a British surgeon who wrote about the surgical treatment of disc displacements in the TMJ. In a groundbreaking article, Costen noted that dental malocclusions contributed to ear and TMJ pain and other ear symptoms, such as ringing in the ear, auditory perception loss, and vertigo. In the following years, no evidence supported the sole-factor theories of TMD, such as the TMJ, muscles, or tooth occlusion.

Etiology of TMDs

TMDs are thought to have a complicated, multifaceted cause. Poorly understood and often misunderstood is the etiology of TMD. No single "cause" exists because there are starting, predisposing, and perpetuating variables [16]. The causes of the condition include joint laxity, exogenous estrogen, bruxism, orthodontic instability, occlusal abnormalities, and micro and macro trauma [17]. Below is a structured version of the information provided, organized in a table format to clearly delineate the predisposing factor, initiating factor, and perpetuating factor (Table 1).

**Table 1:** Etiological factors of temporomandibular disorders (TMDs)

Category	Description
Predisposing factor	Raise the possibility of developing temporomandibular dysfunction. Pathophysiological, psychological, and structural processes that alter the masticatory system.
Initiating factor	It is what causes an illness to start. It is more likely connected to the paradoxical loading and trauma of the musculoskeletal system.
Perpetuating factor	Impede the recovery process or accelerate the development of temporomandibular disorders. Behavioral cause, social cause, emotional cause, and cognitive cause.

*Occlusal Factor* 

The most primary and contentious for the TMDs is occlusion. Clinically speaking, "occlusion" refers to all of the stomatognathic system's components, including the teeth and their supporting tissues, the neuromuscular system, bones, and the TMJ, as well as the dynamic morpho-physiological interactions between them [18]. Occlusion may contribute to susceptibility and the onset or progression of TMD. Nowadays, it is one of the factors that most researchers consider when analyzing TMDs [19]. Below is a structured table summarizing the studies and findings on TMDs as provided (Table 2).

**Table 2:** Results of the previous studies conducted in this field.

Sr No.	Authors	Study	Finding
1.	Pullinger and Selignen [20]	Comparison of occlusal features between patients with and without temporomandibular disorders symptoms	Some occlusal traits may be symptoms rather than causes, and malocclusion may contribute to the development of temporomandibular disorder. Occlusal variables account for 10-20% of temporomandibular disorders etiological factors.
2.	Rammelsberg et al. [21]	Summary of the etiopathogenic theory for temporomandibular disorder emergence	Occlusal instability increases the risk of severe wear and poor therapeutic outcomes in posterior teeth.
3.	Le Bell et al. [22]	Subjective responses to artificial interferences in individuals with and without a history of temporomandibular disorder	Artificial interferences do not cause dysfunctional symptoms in healthy individuals but exacerbate clinical symptoms in those with a history of temporomandibular disorder.
4.	Padala et al. [23]	The connection between temporomandibular disorder signs and symptoms and condylar position and centric occlusion-centric connection discrepancy.	Individuals with temporomandibular disorder may exhibit significant dental inter-arch discrepancies and notable condylar displacements, which can be identified by measuring centric relation-centric occlusion discrepancy.

Psychological

Research in psychology reveals that individuals suffering from TMD share the same psychological makeup and maladjustment as those with other chronic musculoskeletal conditions like arthritic or tension-type back pain [24]. The study conducted by Kindler et al. (“Depressive & Anxiety Symptoms as Risk Factors for Temporomandibular Joint Pain: A Prospective Cohort Study in the General Population") shows that depression and anxiety act as the risk factors. The study found that depressive symptoms had a stronger correlation with joint pain than muscle pain, but anxiety symptoms had a stronger correlation with muscle pain. There are at least two reasons why there might be a connection between depressive or anxious symptoms and a higher chance of experiencing joint or muscle pain. Anxiety and depression may initially manifest as hyperactivity in the muscles, which can subsequently result in anomalies in the muscles and changes in their mechanics, all of which can lead to pain in the muscles [25]. Inflammation of the joints, as well as biomechanical changes, may result from them. In addition, abnormal pain processing in the trigeminal system brought on by imbalances in common neurotransmitters like serotonin and catecholamines may be linked to TMD [26]. Table 3 shows the relationship between psychological factors with TMD.

**Table 3:** Psychological factors and their relationship with TMD TMD: temporomandibular disorder, TMJ: temporomandibular joint

Psychological factor	Relationship with TMD
Stress	Increase in the stress can cause an increase in the somatic reaction, leading to linear increase in TMD [27].
Anxiety	It can intensify the chewing muscle hyperactivity linked to TMJ disorders, which can lead to joint overload. It is frequently associated with TMJ disorder [28].
Depression	It is connected to how severe the pain from the TMD is [29].

Hormones and TMJ

Women are more prone to have TMDs than men. TMDs primarily affect women, which supports the idea that estrogens may play a role in their pathomechanism, peaking at 20 to 45 years of age [30]. Patients with TMD experience painful sensations during the menstrual cycle and during pregnancy due to hormonal changes [31]. Although women of childbearing age are more susceptible to periodontitis and gingiva hyperplasia due to high levels of estrogen during pregnancy, myofascial pain may worsen due to variations in estrogen levels. Conversely, low estradiol levels during menopause are linked to osteoporosis, alveolar bone resorption, and TMJ degeneration [32]. Women with TMD feel less discomfort during pregnancy when there is an increase in progesterone, estrogens, and relaxin hormones that have a significant influence on TMJ degeneration. The only hormone produced during pregnancy, relaxin, increases the flexibility and laxity of the TMJ muscles, tendons, and ligaments [33]. Uncertainty surrounds the path mechanism by which female reproductive hormones affect the masticatory organ. Because it boosts the production of matrix metalloproteinase (MMP9 and MMP13) in the fibrocartilage of the TMJ, which leads to cartilage degradation, 17-estradiol (E2) in women of reproductive age plays a crucial role in the pathogenesis of lesions of the masticatory organs [34]. The mechanisms behind the harmful effects of E2 are, regrettably, poorly understood [35].

Changes in occlusion secondary to TMDs

Anterior Open Bite

According to multiple reports, the most typical malocclusion among TMD patients is an open bite [36]. According to an epidemiological study, the most prevalent bite pattern among TMD patients is anterior open bite [37]. According to a case study report about the ‘’acute anterior open bite,’’ the anterior open bite occurs due to the internal derangement of the TMJ. One more case study conducted by Alzabeg et al. found that after using a soft night guard to treat TMD symptoms, a sudden acute open bite with an undetermined cause occurred.

Association With Unilateral Posterior Bite

Table 4 shows that there has been research on a connection between posterior bite and TMDs. Based on publications chosen by predetermined criteria, the current updated review concurs with past thoughts in finding both a positive and a negative correlation between TMD and posterior cross-bite.

**Table 4:** Results of the previous studies conducted on TMDs and crossbite TMJ: temporomandibular joint, TMDs, temporomandibular disorders; FUPC: functional unilateral posterior crossbite, RCP: retruded contact position, ICP: intercuspal contact position

Sr No.	Author	Year	Finding
1.	De boer and stinks et al. [38]	1997	The findings of this small study suggest that temporomandibular dysfunction cannot be prevented with FUPC treatment.
2.	Egermark I et al. [39]	2003	Other malocclusions have a weak relationship with TMDs except for lateral force between RCP and ICP, unilateral crossbite.
3.	Demir et al. [40]	2005	They found statically that all occlusal factors are related to muscle tenderness except for the posterior crossbite and functional shift.
4.	Farella et al. [41]	2007	A young adolescent's TMJ disk displacement is not associated with unilateral crossbite.

Interestingly, while crossbite type was disregarded, there was general interest in the various TMD variables. This can be seen in subcategories of TMDs, such as fractures, joint discomfort, articular disorder (disc disorders such as acute anterior disc displacement without reduction), joint stiffness (synovial chondromatosis), and masticatory muscle disorder subcategories [42]. Disorders of the muscles of mastication (MMDs) include myalgia or spasms. In sensitive spots within taut muscle bands, these points may signal myofascial pain. Two mild occlusal abnormalities that can cause trigger areas in the inferior lateral pterygoid are disoccluded ipsilateral posterior teeth and premature anterior teeth contact (Table 5).

**Table 5:** Summary of the subcategories of TMDs TMDs: temporomandibular disorders, TMJ: temporomandibular joint, DC-TMD: Diagnostic Criteria for Temporomandibular Disorders

Subcategories of temporomandibular disorders	Definition	Clinical examination
Joint pain	According to the expanded taxonomy of DC-TMD, the condition is defined as TMJ ‘’pain that is not linked to any systemic illness and is accompanied by clinical signs of localized inflammation or infection.’’	Range of motion and palpation of the lateral aspect of the TMJ may replicate the patient's pain complaints. Inflammatory symptoms like redness, swelling, or elevated temperature in the TMJ region may accompany this. One-sided or bilateral posterior open bites with deep anterior tooth contacts may result from intraarticular swelling and effusion of inflammatory exudate, depending on the condition's severity.
Disc disorder	A disc condyle complex-related intracapsular biomechanical disorder is known as disc displacement without reduction with limited opening (DDW/OR). Acute disc displacement without reduction with limited opening cases typically results in anterior and medial articular disc displacement, with posterior and lateral displacement occurring less frequently.	In acute anterior DDW/OR, the disc becomes anteriorly dislodged, and the condyle immediately presses firmly against the methodical tissue. On the affected side, this could result in slightly altered occlusal contact or mild occlusion changes. The retrodiscal tissue may then become irritated, potentially leading to a posterior open bite.
Synovial chondromatosis	Benign lesion with multiple calcifications in the joint as a result of cartilaginous metaplasia in the synovium.	Preauricular swelling and the patient's pain complaints reproducing with TMJ palpation are possible [43].
Fractures	The bony parts of the joint may be affected by a displaced or non-displaced fracture, which may result in occlusal abnormalities like a contralateral posterior open bite.	Preauricular swelling and the patient's pain complaints reproducing with TMJ palpation are possible.

TMDs and occlusion

There has been debate about the function of occlusion in TMDs for many years. An early systematic review published in 2004 examined the relationship between TMDs, malocclusion, and functional occlusion parameters (such as nonworking-side occlusal contacts and occlusal interference). It was discovered that the number of associations was relatively low [44]. The scientific evidence available until that point was insufficient to draw firm conclusions about the relationship between TMD and particular untreated malocclusion, according to a 2005 review conducted by the Swedish Council on Health Technology Assessment [45]. Limited scientific evidence was also available to support the association between orthodontic treatment and TMD. A review carried out in 2007 by Mohlin et al. confirmed these findings [46]. It discovered no link between certain malocclusions or orthodontic procedures and the symptoms of TMD. A limited number of studies found a correlation between anterior spatial relationships and attrition, according to a review on decay, occlusion, and masticatory system dysfunction [47].

A study found that having fewer teeth could result in a higher tooth wear index, but no study found that losing posterior teeth increased attrition; instead, it seemed to be associated with self-identified teeth grinding. However, there was not enough information to make definite judgments about how TMD and wear and tear related. In 2018, a different in-depth review of the positional and dimensional changes in the TMJ after correcting children's posterior crossbites found insufficient data to draw a firm conclusion [48]. The summarized version of the articles that were part of the review is displayed in Table 6.

**Table 6:** Summary of articles included in the review. TMD: temporomandibular disorders, TMJ: temporomandibular joint, FUPC: functional unilateral posterior crossbite, RCP: retruded contact position, ICP: intercuspal position

Authors	Year	Findings
Gauer et al. [1]	2015	About the temporomandibular disorders
Manfredini et al. [2]	2017	There needs to be more evidence to support a more extensive theory that dental occlusion contributes to the pathophysiology of temporomandibular disorder.
Henrikson et al. [3]	1997	Normal occlusion has lower signs and symptoms than class 2 occlusion, which has increased signs and symptoms from the other groups.
McNeill et al. [4]	1990	Temporomandibular disorder diagnosis management and education and research.
Gesch et al. [5]	2004	Women showed higher signs and symptoms than men.
Suvinen et al. [6]	2005	Variations in the psychosocial variables determine how temporomandibular disorders manifest and are subtyped.
McNeill et al. [7]	1997	Temporomandibular disorder guidelines.
Visscher et al. [8]	2009	In order to verify a suspicion of temporal bone pain, positive dynamic/static tests are preferable. A negative RDC/TMD examination is a better indicator of the absence of TMD pain.
Glaros et al. [9]	1994	The etiology of temporomandibular disorders is commonly recognized by practicing dentists to involve mental health disorders and psychophysiological factors.
Luther et al. [10]	2007	There is insufficient data to support the theory that static occlusal factors cause TMD.
List et al. [11]	2010	Management of temporomandibular disorder.
Macfarlane et al. [12]	2009	TMD is neither caused nor prevented by orthodontic treatment. The only factors that could predict TMD in early adulthood were female sex and TMD during adolescence.
Schiffman et al. [13]	2012	Diagnostic criteria for headache secondary to temporomandibular disorders (TMDs).
LeResche et al. [14]	1997	Additionally, it appears that women experience the majority of the symptoms and indicators linked to specific temporomandibular disorders more frequently than men.
Costen et al. [15]	1934	The symptoms of ear and sinus disorders depend on the disturbed function of the temporomandibular disorder.
McNeill et al. [16]	1997	The majority of treatment for temporomandibular disorder would be non-invasive techniques that provide relief.
Gage et al. [17]	1985	Early identification of non-painful clicking in young adults TMJs may shield the condyle, disk, and temporal bone complex from long-term harm.
Türp et al. [18]	2008	Dental occlusion about the past, present, and future.
McNeill et al. [19]	1990	Craniomandibular guidelines.
Pullinger et al. [20]	2000	Though they might play a part, occlusal factors should be balanced in the identification of the TMD.
Chisnoiu et al. [21]	2015	Inadequate knowledge of temporomandibular disorders etiology.
Bell et al. [22]	2002	Patients with a history of temporomandibular disorder experience worsening clinical symptoms due to artificial interferences.
Padala et al. [23]	2012	Measuring and analyzing the centric relation centric occlusion discrepancy can reveal significant condylar displacements and dental inter-arch discrepancies in individuals with TMD.
Suvinen et al. [24]	1997	Typical forms of temporomandibular disorders.
Scrivani et al. [25]	2008	Common forms of temporomandibular disorders.
Bair et al. [26]	2003	In a pain sample, the prevalence of depression is higher than the prevalence rates when the conditions are looked at separately.
Pingitore et al. [27]	1991	This study shows that both physical and psychological factors are associated with bruxism.
Nguyen et al. [28]	2019	There was no correlation found between anxiety, depression, or reduced mandibular function and TMJ osseous changes.
Auerbach et al. [29]	2001	The conclusion that psychological factors are more prominent when pain is muscular is primarily supported by prior research showing a connection between emotional dysfunction and TMD.
Kim et al. [30]	2015	There may be physiological and pathological gender differences in TMD, and chronic illnesses and psychological factors play a significant role in chronic TMD.
Wang et al. [31]	2006	E2 upregulates MMP9 and MMP13 on fibrocartilage matrix turnover with a focus on the TMJ.
Robinson et al. [32]	2020	A summary of the TMJ homeostasis, alveolar bone remodeling, and pregnancy-related gingivitis signaling pathways in vitro and in vivo.
Holt et al. [33]	1994	Bacteriology.
Hashem et al. [34]	2006	Overly accelerated deterioration of collagen and GAGs by relaxin and β-estradiol may disturb the ECM's homeostasis in these joints and ultimately aggravate the target joint's degenerative condition.
Kapila et al. [35]	2009	Hormone-mediated induction of particular MMPs to target tissue turnover of the cartilage of specific joints.
Williamson et al. [36]	1977	Percentage of patients at risk for temporomandibular dysfunction who were diagnosed as teenagers before receiving orthodontic treatment.
Egermark et al. [37]	2000	Orthodontic treatment reduces the signs and symptoms of the temporomandibular disorder.
Mohlin et al. [38]	1997	The findings of this small study suggest that temporomandibular dysfunction cannot be prevented with FUPC treatment.
Egermark et al. [39]	2003	Other malocclusions have a weak relationship with TMDs except for lateral force between R.C.P. and I.C.P., unilateral crossbite.
Demir et al. [40]	2005	They found statically that all occlusal factors are related to muscle tenderness except for the posterior crossbite and functional shift.
Farella et al. [41]	2007	A young adolescent's TMJ disk displacement is not associated with unilateral crossbite.
Van't Spijker et al. [42]	2007	There is limited knowledge available about the relationship between TMD symptoms and attrition.
Padala et al. [43]	2012	The condyle position may significantly influence the etiopathogenesis of TMJ disorders.
M. De Boer et al. [44]	2008	FUPC should be treated early in order to achieve average growth and development rather than to prevent temporomandibular disorders.
Demir et al. [45]	2005	Female subjects had a higher prevalence of temporomandibular disorders, which their more incredible masticatory muscle tenderness may explain.
te Veldhuis AH et al. [46]	2011	Synovial chondromatosis is one of the differential diagnoses of the temporomandibular disorder.
Gary et al. [47]	2022	Insufficient investigation into the temporomandibular joint and occlusion.
Thomas et al. [48]	2023	This is a modern interpretation of temporomandibular dysfunction.

## Conclusions

The correlation between occlusion and TMDs is multifaceted and intricate. It is crucial to understand that TMD is a condition influenced by many factors, even though there is evidence suggesting that occlusion may contribute to symptoms. These are some examples of occlusal disparities, tense muscles, stress, trauma, and other unique factors. Occlusal discrepancies are not the only cause of TMD cases, as several different variables, such as muscle tension, stress, trauma, and individual differences in anatomy and physiology, also impact the disorder. Pain, anxiety, physical limitation, and limited mandibular movement are some of the symptoms of TMDs, which can progressively worsen and decrease the level of comfort. There are few alternatives to therapy available, and sometimes these are insufficient to meet the long-term needs of the relatively young patient population. Because of this, it is crucial to identify potential etiologic factors as soon as possible, along with how much they may be affecting the condition. Then and only then can the best course of action to relieve the incapacitating TMD symptoms be determined.
